# Local staging and treatment in extremity rhabdomyosarcoma. A report from the EpSSG‐RMS2005 study

**DOI:** 10.1002/cam4.3365

**Published:** 2020-09-01

**Authors:** Sheila E. J. Terwisscha van Scheltinga, Marc H. W. A. Wijnen, Hélène Martelli, Timothy Rogers, Henry Mandeville, Mark N. Gaze, Keiran McHugh, Nadege Corradini, Daniel Orbach, Meriel Jenney, Anna Kelsey, Julia Chisholm, Soledad Gallego, Heidi Glosli, Andrea Ferrari, Ilaria Zanetti, Gian Luca De Salvo, Veronique Minard‐Colin, Giani Bisogno, Max M. van Noesel, Hans H. M. Merks

**Affiliations:** ^1^ Pediatric Surgery Pediatric Solid Tumor Unit Princess Máxima Center for Pediatric Oncology Utrecht The Netherlands; ^2^ Department of Pediatric Surgical Oncology University Hospital Bicětre Bicětre France; ^3^ Department of Pediatric Surgery University Hospitals Bristol NHS foundation trust Bristol UK; ^4^ Children and Young People's Unit Royal Marsden Hospital Sutton UK; ^5^ Department of Oncology Great Ormond Street Hospital for Children London UK; ^6^ Department of Radiology Great Ormond Street Hospital for Children London UK; ^7^ Institut d’Hématologie et d'Oncologie Pédiatrique Centre Léon Bérard Lyon France; ^8^ SIREDO Oncology Center Institut Curie PSL University Paris France; ^9^ Department of Pediatric Oncology University hospital of Wales Cardiff UK; ^10^ Department of Pathology Central Manchester University Hospitals Manchester United Kingdom; ^11^ Children and Young People's Department Royal Marsden Hospital Sutton United Kingdom; ^12^ Pediatric Oncology Vall d'Hebron University Hospital Barcelona Spain; ^13^ Division of Pediatric and Adolescent Medicine Department of Pediatric Research Oslo University Hospital Oslo Norway; ^14^ Pediatric Oncology Unit Fondazione IRCCS Istituto Nazionale dei Tumori Milano Italy; ^15^ Clinical Trials and Biostatistics Unit IRCCS Istituto oncologico Veneto Padova Italy; ^16^ Department of Pediatric and Adolescent Oncology Gustave Roussy Villejuif France; ^17^ Hematology and Oncology Division Department of Women’s and Children’s Health Padova University Hospital Padova Italy; ^18^ Pediatric Solid Tumor Unit Princess Maxima Center for pediatric Oncology Utrecht The Netherlands

**Keywords:** local therapy, lymph node metastases, radiotherapy, rhabdomyosarcoma, staging, surgery

## Abstract

Rhabdomyosarcoma of the extremities present with two main challenges: correct evaluation of initial regional nodal involvement and define adequate local treatment.

**Methods:**

Pediatric patients with localized rhabdomyosarcoma of the extremity included in the EpSSG‐RMS2005 study between 2005 and 2014 were evaluated for staging, treatment, and survival. The outcome was compared to the preceding European SIOP‐MMT studies.

**Results:**

Of the 162 patients included, histology was unfavorable in 113 (70%), 124 (77%) were younger than 10 years, 128 (79%) were IRS III, and 47 (29%) were node‐positive. A regional node biopsy was performed in 97 patients (60%) and modified the lymph node stage in 15/97 (16%). Primary and delayed surgery was performed in 155 (96%) and radiotherapy delivered in 118 (73%) patients. Relapse occurred in 61 cases (38%), local in 14 (23%), regional in 13 (21%), distant in 22 (36%), and combined relapse in 12 (20%) with five progressive diseases (8%) and four secondary tumors (7%). Five‐year event free (EFS) and overall survival (OS) were 58.4% (95%CI, 50.3‐65.7) and 71.7% (63.6‐78.4), respectively. In the previous studies MMT89 and MMT95, tumor surgery was performed in 32/53 (60%) and 74/82(90%), respectively, and radiotherapy delivered in 13/53 (25%) and 26/82 (30%), respectively. Five‐year EFS and OS were 35.6%, and 50.3% in MMT89 and 54.3% and 68.2% in the MMT95 study.

**Conclusions:**

Even if the lymph node staging was not always complete according to the RMS2005 protocol, node sampling changed lymph node status in a significant number of patients. Despite the higher rate of patients treated with locoregional radiotherapy, survival in RMS2005 did not improve compared to the previous European SIOP‐MMT95 study.

## Graphical abstract

Adequate staging is important for the treatment of extremity RMS. Despite local therapy intensification, survival in RMS2005 did not improve compared to the previous European SIOP‐MMT95 study.

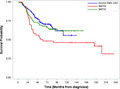

## 1 INTRODUCTION

Rhabdomyosarcomas (RMS) of the extremities in children account for approximately 15% of RMS and have overall a poor outcome.^1^, ^2^, ^3^, ^4^ Several adverse factors are frequently associated with extremity RMS, including older age, alveolar subtype (ARMS), and nodal involvement.^4^ Lymph node metastases are present in 20%‐50% of patients at diagnosis.^4^, ^5^ Approximately 33%‐43% of the patients with extremity RMS relapse in the regional nodes with an inherent poor salvage rate (9%‐32%).^4^, ^6^ Efforts should be made to improve initial regional staging and loco‐regional therapy in extremity RMS.

We evaluated the impact of staging and local treatment in localized extremity rhabdomyosarcoma and determined its influence on outcome in the European EpSSG‐RMS2005 study.

## 2 PATIENTS AND METHODS

Pediatric patients with localized RMS were prospectively enrolled in the EpSSG‐RMS2005 study.^7^, ^8^ For this analysis, patients diagnosed between September 2005 and December 2014 were included to ensure at least a 3‐year follow‐up. All participating centers were required to obtain written approval from their local authorities and ethical committees, as well as written informed consent from patients or their parents or legal guardians.

The extremity site was defined as any part of the upper and lower extremity, buttock, and shoulder girdle. The distal site was defined as foot, ankle, and leg in the lower extremity, and as hand, wrist, and forearm in the upper extremity.

Regional lymph node involvement was defined as clinical, radiological, and/or pathological tumor deposits in lymph nodes or lymph vessels between the primary tumor and the regional lymph node basin (groin or axilla) and regarded as a localized disease. Lymph node metastases beyond the regional basin or in mediastinum were marked as metastatic disease and therefore not included in this study.

### 2.1 Diagnosis and staging

Primary histology was provided by open or trucut biopsy; histopathological material was centrally reviewed. Molecular confirmation of the presence of a FOXO1 translocation was recommended, but not mandatory to classify a tumor as alveolar. Histology was classified as unfavorable when the histology was alveolar or a translocation was present in not otherwise specified histology. A biopsy of the regional nodes was mandatory, even if not suspicious of clinical and radiological examination. The sentinel node technique was recommended whenever feasible.

The primary radiologic assessment was performed by MRI, ultrasound, and/or CT. In lower extremity tumors, additional abdominal and pelvic metastases were investigated by CT or MRI. Chest CT was used to exclude pulmonary metastases. Bone marrow aspirations and biopsies were mandatory. When the study started a Technetium bone scan was mandatory; this was gradually replaced by FDG‐PET‐CT. Original radiology reports were reviewed for all patients; central imaging review was not part of this study. Patients were stratified in risk groups according to defined risk factors (Table S1).^8^, ^9^

### 2.2 Study treatment

Depending on the risk group (Table A1), patients were treated with VA (vincristine, actinomycin‐D; low‐risk group), IVA (ifosfamide + VA; standard risk) or randomized to IVA vs IVADo (IVA + doxorubicin; high risk).^7^ Patients in the high‐risk group, in clinical remission after standard chemotherapy, were randomized to stop therapy or receive maintenance chemotherapy (6 × 28‐day cycles of low‐dose oral daily cyclophosphamide + weekly intravenous vinorelbine).^8^ Patients with alveolar RMS and lymph node involvement received systematically IVADo followed by maintenance therapy (very high risk).^8^, ^10^

Upfront resection of the local tumor was only performed if an excision with adequate margins and without mutilation seemed possible. Completeness of initial surgical resection was reported using IRS postsurgical stage.^11^

Local treatment after four cycles of initial chemotherapy included delayed surgery and/or radiotherapy when indicated. Delayed surgery aimed to provide an adequate margin but, as a rule, was conservative, anticipating radiotherapy for residual disease. However, extensive surgery was considered in very young children if radiotherapy could be prevented.

Local radiotherapy was recommended for all alveolar patients, those with IRS group II and III diseases, and mandatory for patients in the high‐risk category. Radiotherapy with doses of 36.6, 41.1, and 50.4 Gy was given to the primary tumor depending on histology, IRS stage, tumor response, and completeness of delayed resection. Radiotherapy (41.4 Gy) to the regional nodes was performed in patients with clinical, radiological, or pathological node involvement. An additional boost of 9 Gy was applied in cases with residual disease after chemotherapy and surgery.

### 2.3 Comparison with previous SIOP‐MMT studies

To evaluate the efficacy of the EpSSG‐RMS2005 protocol, we compared treatment, relapse rate, and survival with data from the International Society of Pediatric Oncology Malignant Mesenchymal Tumor Committee (SIOP‐ MMT) 89 and 95 protocols.^6^, ^12^ In the MMT89/95 studies, patients were treated with chemotherapy using (I)VA, or an anthracycline‐based “6‐drug” regimen. Primary surgery was encouraged when achievable without leaving residual disease and functional impairment. Delayed surgery was conservative. Radiotherapy to the primary tumor was applied when complete remission could not be achieved with chemotherapy, with or without surgery. Only from 2001 on patients older than 3 years with alveolar histology were to receive standard radiation therapy. Lymph nodes were only to receive radiotherapy when still enlarged after chemotherapy.

### 2.4 Endpoints and statistics

Statistical calculations were made using SAS 9.4 (SAS Institute Inc, Cary, NC, USA). Five‐year event‐free survival (EFS) was calculated from the date of diagnosis, up to the time of a first event, which was defined as a relapse, progression, secondary tumor or death. Overall survival (OS) was calculated from the date of diagnosis up to the death of any cause. The survival curves were estimated by Kaplan‐Meier analysis.^13^ A Cox proportional hazard analysis was performed to evaluate predicting variables such as age, gender, tumor size, site, nodal status, tumor invasiveness, postsurgical status, histology, fusion status, and radiological staging modality. Survival was compared to SIOP‐MMT study 89 and 95 (MMT89/95) ^6^, ^12^ using the Kaplan‐Meier method.

## 3 RESULTS

We included 162 patients with nonmetastatic RMS of the extremities. Patient and tumor characteristics are listed in Table 1; there was an equal distribution according to gender, most patients were under 10 years of age (76.5%), had tumor size >5 cm (64.2%), unfavorable histology (69.8%), were IRS III postsurgical stage (79.0%), had T1 stage (77.8%), lymph node involvement (29.0%), and tumors of the lower extremities (61.7%).

**TABLE 1:** Patient and tumor characteristics and influence on event‐free (EFS) and overall survival (OS)

		No of patients (N = 162)		%	5 year‐EFS (95%CI)	*P*‐value	5 year‐OS (95%CI)	*P*‐value
Gender								
Male		78	48.2		58.3 (46.5‐68.5)	.5358	68.1 (55.4‐77.9)	.5061
Female		84	51.8		58.7 (47.1‐68.5)		74.9 (63.7‐83.1)	
Age (year)								
<3		54	33.3		71.9 (57.8‐82.0)	.0741	82.2 (68.5‐90.4)	.0619
3‐9		70	43.2		49.7 (37.0‐61.1)		71.6 (58.4‐81.2)	
10‐18		38	23.4		55.0 (38.0‐69.2)		57.9 (39.9‐72.3)	
Tumor size								
≤5 cm		58	35.8		66.4 (52.3‐77.1)	.0910	81.5 (68.3‐89.7)	.1166
>5 cm		104	64.2		53.9 (43.7‐63.1)		66.2 (55.4‐74.9)	
Histology								
Favourable		49	30.2		76.8 (62.0‐86.5)	.0072	87.0 (73.2‐94.0)	.0174
Unfavourable		113	69.8		50.5 (40.8‐59.4)		65.3 (55.0‐73.7)	
Fusion status (for unfav. histology)		(113)						
FOXO1 fusion present, type not known		26	23		51.2 (39.7‐61.5)	.9775	63.1 (50.6‐73.2)	.4251
PAX3		40	36.7					
PAX7		15	13.8					
Negative		15	13.8		51.4 (23.8‐73.5)		86.7 (56.4‐96.5)	
Not performed		17	14.7					
IRS stage								
I (R0/N0)		14	8.6		78.6 (47.2‐92.5)	0.0169	85.1 (52.3‐96.1)	0.0480
II (R1 and N0/N1)		20	12.4		79.3 (53.7‐91.7)		95.0 (69.5‐99.3)	
III (R2 and N0/N1)		128	79.0		53.0 (43.8‐61.3)		66.1 (56.4‐74.2)	
T stage								
T1(confined to tissue of origin)		126	77.8		64.3 (55.1‐72.1)	0.0021	78.5 (69.8‐85.0)	0.0007
T2 (beyond tissue of origin)		36	22.2		37.6 (21.9‐53.3)		45.5 (26.4‐62.7)	
N‐stage								
N0 (no lymph node metastases)		115	71.0		64.5 (54.8‐72.6)	0.0274	80.3 (71.2‐86.7)	0.0034
N1 (lymph node metastases)		47	29.0		43.2 (28.6‐57.0)		50.4 (34.2‐64.6)	
Primary tumor site								
Lower extremities		100	61.7		63.5 (53.1‐72.1)	0.2549	77.7 (67.4‐85.1)	0.1321
Upper extremities		62	38.3		50.4 (37.1‐62.3)		62.1 (48.1‐73.3)	
Distal tumor		97	59.9		55.1 (44.6‐64.4)	0.3148	66.9 (55.7‐75.9)	0.3175
Proximal tumor	65		40.1		63.3 (50.0‐74.0)		78.7 (66.0‐87.1)	

Abbreviations: R, surgical resection margin; N, lymph node status; CI, confidence interval.

### 3.1 Staging

Biopsy of the primary tumor was performed in 122/162 patients (75.3%), needle in 40, and incisional biopsy in 82 patients. The 40 remaining patients had 36 immediate primary resections and in four, histological diagnosis was made by regional lymph node biopsy only. In 58/162 (35.8%) patients, an FDG‐PET‐CT was performed.

**FIGURE 1:**
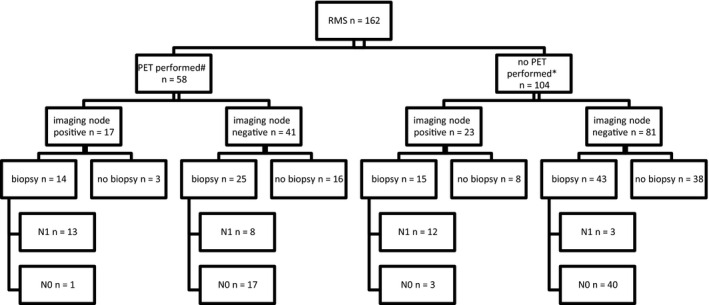
Flowchart showing lymph node staging by radiology and surgery/histology. *Only standard imaging by MRI, CT, and ultrasound. # PET in addition to standard imaging. N1: lymph node metastases; N0: no lymph node metastases

Clinical and radiological imaging staged 40 patients as node‐positive and 122/162 as node‐negative. When a combination of FDG‐PET‐CT, in addition to standard imaging, was compared to standard imaging, lymph node metastases were suspected in 29.3% (17/58) of patients compared to 22.1% (23/104) for the group without FDG‐PET‐CT (difference not significant [*P* = .31]; Figure 1). Lymph node biopsy changed the nodal status from “suspected to be positive” to “negative” in 4/29 patients and from negative suspicion to positive in 11/68 patients, changing overall clinical/radiological lymph node status in 15.5% of the nodal biopsied tumors (Figure 1).

Lymph‐node sampling at diagnosis was performed in 39/58 (67.2%) of FDG‐PET‐CT staged patients and in 58/104 (55.8%) of the other patients. FDG‐PET‐CT was false‐positive in 1/14 (7.1%) and false‐negative in 8/25 (32.0%) patients, leading to a sensitivity of 61.9% and specificity of 94.4%. In these eight false‐negative cases, a sentinel node biopsy was performed in 4 and node picking in 4. In patients not staged with FDG‐PET‐CT, classical radiology imaging had false‐positive results in 3/15 (20.0%) patients and false‐negative results in 3/43 (7.0%), leading to a sensitivity of 80.0% and specificity of 93.0%. In those three false‐negative patients, no sentinel node procedures were performed. In 65/162 (40.1%) of patients, no biopsy of the nodes was performed.

Overall 47/162 (29.0%) patients were considered to have definitive regional lymph node involvement and treated accordingly; clinically and radiologically in 11/47 and histologically proven in 36/47 patients.

### 3.2 Chemotherapy

Patients were treated according to their risk stratification; four patients were low risk, 15 standard risk, 103 patients high risk, and 40 patients very high risk. Most patients received either IVA (Do) without maintenance therapy (81/162) or with maintenance therapy (69/162). Three patients had VA regimen, two IVA‐VA, and seven patients started with IVA(Do) but switched to other combinations of chemotherapy.

### 3.3 Local therapy

#### 3.3.1 Initial tumor resection

Primary resection was performed in 36/162 patients, with an immediate re‐resection in 10/36 patients. There were macroscopic residuals (R2 resection) in three patients, microscopic residuals (R1) in 15, and complete resection (R0) in 18.

#### 3.3.2 Delayed surgery

Among the 128 IRS III patients, 47 were lymph node‐positive; primary site was proximal arm in 12, distal arm in 37, proximal leg in 38, and distal leg in 41. Ninety‐three percent (119/128) underwent delayed surgery. Of the remaining nine patients, surgery was not performed due to early progression in three complete remission (CR) after chemotherapy in five patients, and therefore only RT was delivered and expected mutilating surgery in one patient after a partial response (local therapy: RT only). Among 116/119 patients for which detailed surgical treatment data were available, 44 patients underwent tumorectomy, 55 wide excisions, 15 amputations, and in 2 patients only a biopsy for histological verification was performed. A complete resection (R0) was obtained in 87/116 (75.0%), microscopic residuals (R1) in 23, macroscopic residuals (R2) in 3, and no tumor was found in 3.

### 3.4 Radiotherapy

Locoregional RT was delivered in 118/162 patients (72.8%). The primary tumor was irradiated in 76/118, primary tumor and nodes in 39, only the nodes in 3 (all patients with limbs amputation). In 44 cases, local RT was not delivered because of low/standard risk tumor in 6, young age (<3 years) in 15, radical surgery/amputation in 10, center decision/parental refusal in 8, and early progressive disease in 5 patients.

External beam RT was delivered in 108/118 patients, brachytherapy in 6, and a combination in 4. The median dose to the primary tumor was 41.4 Gy (IQR 41.4‐50.4).

Of the 47 N1 patients, 40 had RT to the nodes. An amputation was performed in 1/47 N1 patients including the involved nodes. No information was available about the other 6 N1 patients who did not receive RT. In addition, two N0 patients received nodal irradiation for center decision.

### 3.5 Combined local treatment

**FIGURE 2:**
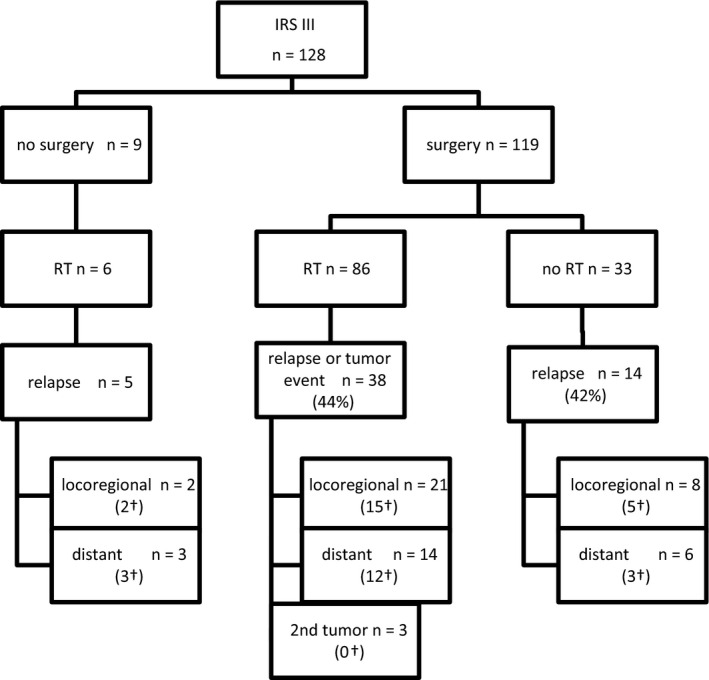
Local therapy in IRS III patients and relapse patterns. ^†^Patients who died; RT radiotherapy

Local treatment of all 128 IRS III patients is listed in Figure 2 and focused on N1 patients in supplemental Figure S1. In addition, three patients had no local therapy because of early progression. In 33 patients, exclusive surgery was performed, 6 patients had definitive RT, and 86 patients had both. Among the 33 patients with exclusive surgery, 27 had an R0 resection, 3 an R1 resection, and 3 an R2 resection.

Of patients with exclusive RT, five had a very good or complete response after chemotherapy and in one, surgery was not performed because of expected mutilating surgery.

### 3.6 Survival and relapse

**FIGURE 3:**
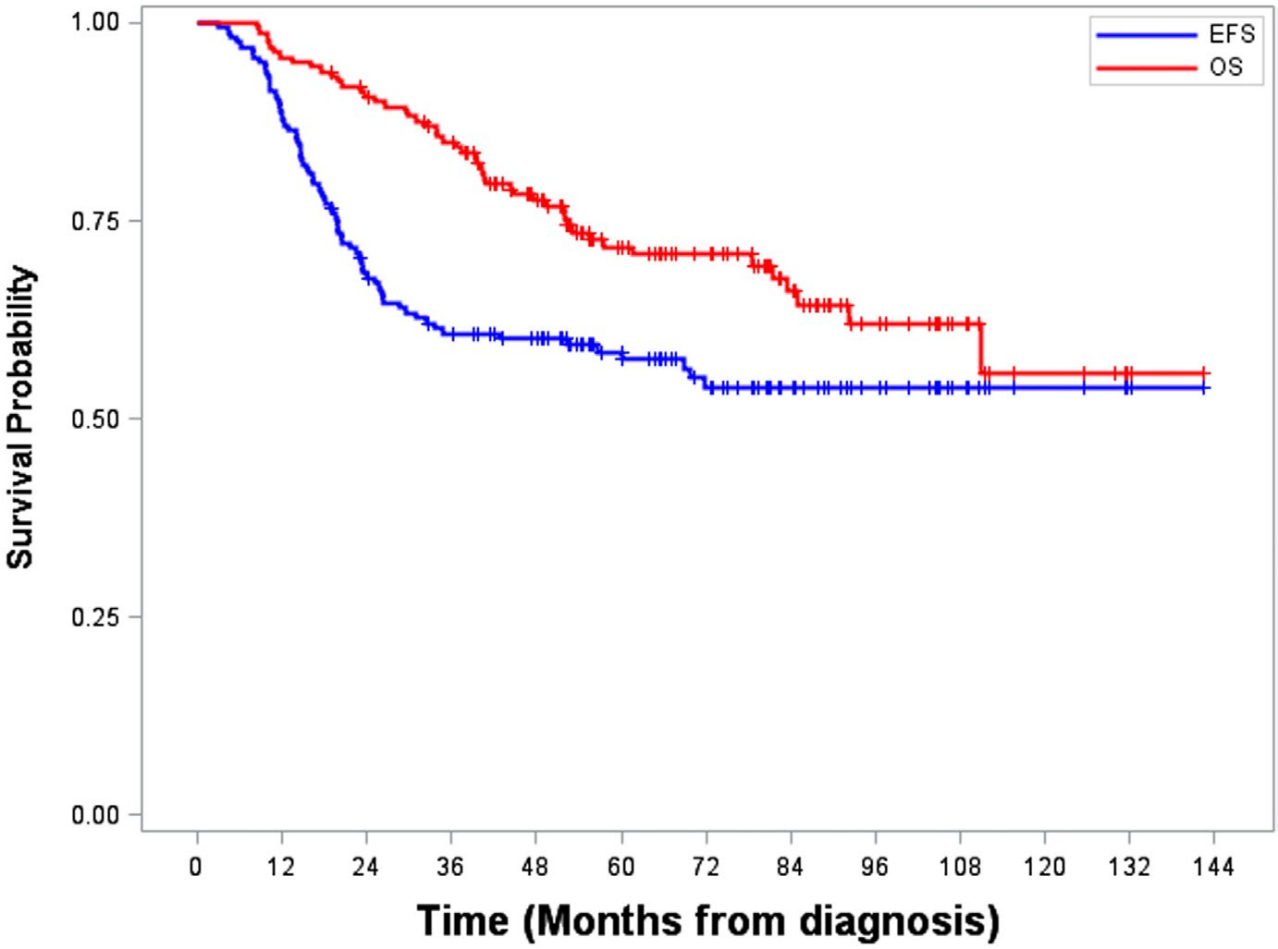
Event Free Survival (EFS) and Overall Survival (OS) – All patients (n = 162)

The median follow‐up of surviving patients was 67.6 months (range 51.8‐88.9). Events occurred in 70/162 (43.2%) patients: local relapse in 14, isolated regional in 13, metastatic in 22, combined in 12 (local/distant (2), local/regional (4), regional/distant (3) or local/regional/distant (3)); in addition, 5 had progressive disease and 4 developed a secondary tumor (3 bone tumors, 1 glioma). Of the 23 patients with a regional or combined (regional in combination with local or metastatic) relapse, 13 (57%) had no lymph node metastases at diagnosis and 10 (43%) were N1. After relapse, 15/70 (21.4%) patients reached a second complete remission; 4/14 (28.6%) after exclusive local relapse, 1/13 (7.7%) after regional relapse, 4/22 (18.2%) after distant, 2/12 (16.7%) after combined relapse, 2/5 after progressive disease. Five‐year event‐free (EFS) and overall survival (OS) for all patients were 58.4% (95%IC 50.3‐65.7) and 71.7% (95%IC 63.6‐78.4), respectively (Figure 3).

### 3.7 Patient and tumor characteristics and survival

Tumor invasiveness, lymph node involvement, unfavorable histology, and high IRS stage were adverse prognostic factors for EFS and OS in univariate analysis (Table 1). Also not performing a biopsy of the lymph nodes in N1 patients had an adverse effect on survival (*P* = .0021).

EFS cox model with significant variables (*P* < .25) in univariate analysis (loco‐regional N, age at diagnosis, size, IRS, primary tumor invasiveness, histology (fav/unfav)), showed that age > 10 years at diagnosis (*P* = .047), positive lymph node status (*P* = .044), and IRS III stage (*P* = .007) had an adverse effect on EFS. Lymph node involvement (*P* = .007) and tumor invasiveness (*P* = .003) had an adverse effect on OS in multivariate analysis, using variables loco‐regional N, age at diagnosis, size, IRS stage, primary tumor invasiveness, site (lower/upper), and histology (fav/unfav).

### 3.8 Local treatment, survival, and relapse in EpSSG‐RMS2005, comparison with previous SIOP‐MMT 89/95 studies

In the SIOP‐MMT studies, 82 patients with localized extremity RMS were included in the SIOP‐MMT 95 and 53 patients in the SIOP‐MMT 89 study. Tumor resection (initial and delayed) was performed in 155/162 (96%) cases in the EpSSG‐RMS2005, in 74/82 (90%) in SIOP‐MMT95, and in 32/53 (60%) in SIOP‐MMT89. Local radiotherapy was delivered in 118/162 (72.8%), 26/82 (30%) and 13/53 (25%), respectively (*P* < .0001), due to different indications for radiotherapy in the different studies (stated in the methods section).

Tumor characteristics, treatment, and survival in the different studies are listed in table 2. Salvage after relapse was 28% in the EpSSG‐RMS2005 study, 20% in SIOP‐ MMT95, and 22% in SIOP‐MMT89.

**TABLE 2:** Tumor characteristics, treatment, and survival in patients included in the EpSSG RMS2005 and MMT 89/95 studies

	MMT89 (N = 53)	MMT95 (N = 82)	EpSSG RMS2005 (N = 162)
Size			
<5 cm	20 (37.7)	26 (31.7)	58 (35.8)
>5 cm	33 (62.3)	56 (68.3)	104 (64.2)
Histology			
Alveolar	30 (56.7)	52 (63.4)	108 (66.7)
Non alveolar	23 (43.3)	30 (36.6)	54 (33.3)
IRS group			
I	8 (15.1)	6 (7.3)	14 (8.6)
II	14 (26.4)	17 (20.7)	20 (12.4)
III	31 (58.5)	59 (72)	128 (79)
Surgery total	32 (60.4)	74 (90.2)	155 (95.7))
Initial	8 (15.1)	28 (34.1)	36 (22.2)
Delayed	24 (45.3)	46 (56.1)	119 (73.5)
Radiotherapy	13 (24.5)	26 (31.7)	118 (72.8)
Relapse			
locoregional	15 (28.3%)	16 (19.5%)	31 (19%)
Metastatic	15 (26.4%)	10 (12.2%)	22 (14%)
Combined	5 (9.4%)	6 (7.3%)	8 (11%)
EFS (5 years)	35.6% (23.0‐48.4)	54.3% (42.8‐64.4)	58.4% (95% CI 50.3‐65.7)
OS (5 years)	50.3% (36.1‐62.9)	68.2% (56.9‐77.1)	71.7% (63.6‐78.4)

Abbreviations: EFS, event free survival; OS, overall survival.

**FIGURE 4:**
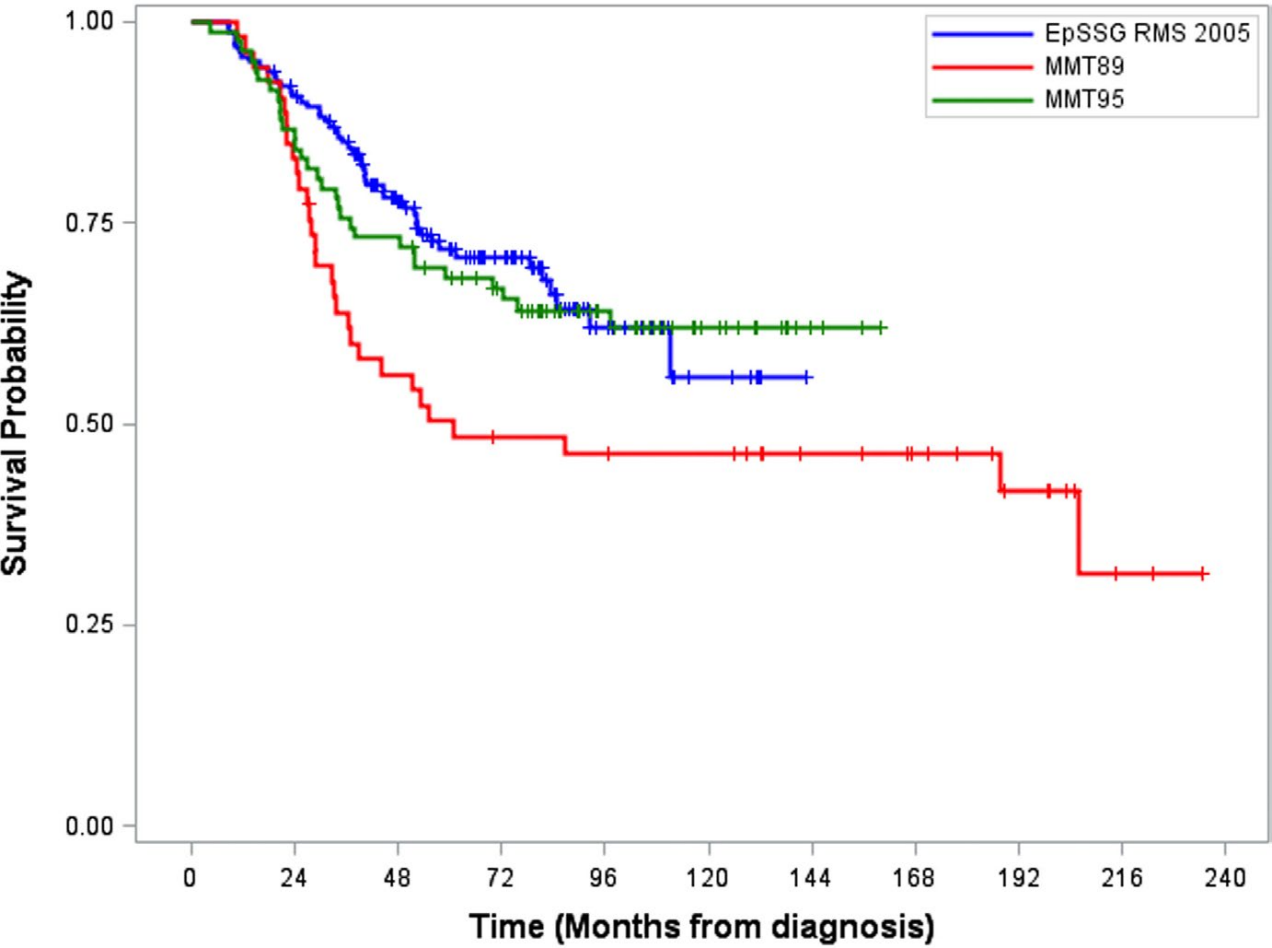
Comparison of overall survival (OS) in the EpSSG 2005, SIOP‐MMT89 and SIOP‐MMT95 studies (*P* = .0384)

The 5‐year‐EFS and OS were 54.3% (42.8‐64.4) and 68.2% (56.9‐77.1) in SIOP‐ MMT95 and 35.6% (23.0‐48.4) and 50.3% (36.1‐62.9) in SIOP‐MMT89 which is significantly different (*P* = .038; Figure 4).

### 3.9 Discussion

In this study, we describe the staging, treatment, and survival of extremity RMS patients treated in the EpSSG‐RMS2005 study.

Overall the 5‐year OS was 72% (95%IC 63.6‐78.4), which is comparable to SIOP‐ MMT95 results. In a pooled analysis, Oberlin et al analyzed patients with extremity RMS (n = 643) from the North American and the European studies treated between 1983 and 2004 and showed a 5‐year overall survival of 67% (standard error 1.8),^4^ which seems comparable to our results. Locoregional and metastatic relapse rates in EpSSG‐RMS2005 were comparable to the MMT95 study. Salvage rates after relapse were poor, especially after a metastatic or combined relapse. Tumor surgery was performed in 93% of IRS III patients in EpSSG‐RMS2005, which was comparable to SIOP‐MMT95 (tumor resection in 90%). When comparing these studies, the most significant improvements in survival and relapse rates were found between the SIOP MMT95 and MMT89 study, where primary surgery was performed more often in MMT95 (90% vs 60% in MMT89), even if the delivery of radiotherapy between studies was comparable (30% in MMT95% vs 30% in MMT89). This may suggest an important role for surgery in disease control. Local therapy was intensified in the EpSSG‐RMS2005 study with 73% of patients receiving radiotherapy, which represents an important increase when compared to the SIOP MMT95 and 89 study. Nevertheless, intensification of the radiotherapy rate did not translate to a decrease in the locoregional relapse rate (19.1% vs 19.5%) neither improve survival (OS 72% (95%IC 63.6‐78.4) vs 68% (95%IC 56.9‐77.1) when compared to the SIOP‐MMT95 study. To be able to explain these results, quality assurance of local therapy will be a part of the next EpSSG study.

In the EpSSG‐RMS2005 study, FDG‐PET‐CT was gradually introduced and so far, not leading to significantly higher positive lymph node detection compared to conventional radiology techniques. However, we found a high false‐negative rate for FDG‐PET‐CT of 32%; this can be explained by the fact that four of eight false‐negative patients had a sentinel node biopsy as part of their systematic work‐up, leading to the detection of micrometastases impossible to detect using FDG‐PET‐CT. In the patients not staged with FDG‐PET‐CT, no sentinel node biopsy was performed in false‐negative patients.

Definitive regional lymph node involvement was diagnosed in 29% of patients with limbs RMS primary. However, only in 60% of the patients histological staging of the nodes was performed according to the protocol. Regional lymph node biopsy changed lymph node status in 16% of patients. This corresponds to the number Neville et al found in clinical and radiological negative patients with limbs RMS (8/46 (17%)).^11^ Lobeck et al noticed an increase in survival in older male extremity RMS patients who underwent a lymph node biopsy in the SEER database.^14^ In our study, a lymph node biopsy showed a survival benefit in N1 patients. Possibly the obviously involved nodes on radiology (containing a higher tumor‐load) were a reason not to biopsy. Four out of 29 (14%) of the radiological positive nodes showed no tumor when a biopsy was performed. Therefore, we strongly recommend to confirm any suspicious nodal enlargement and otherwise to systematically biopsy regional lymph nodes at diagnosis, irrespective of the radiological nodal status. This can be performed by sentinel node biopsy whenever possible or by random node sampling.

For the next EpSSG, Frontline and Relapsed‐Rhabdomyosarcoma (FaR‐RMS) study patients will have radiotherapy quality assurance before starting radiotherapy.^15^ Patients receiving a combination of radiotherapy and surgery will be randomized to pre or postoperative radiotherapy, and patients at high risk for local relapse will be eligible for a radiotherapy dose‐escalation study.

Accurate lymph node staging, prospective radiotherapy plan quality assurance, and delayed surgery when feasible, might improve survival in patients with extremity rhabdomyosarcoma.

## CONFLICT OF INTERESTS

There were no conflicts of interest for all authors.

## AUTHORS CONTRIBUTION

Sheila Terwisscha van Scheltinga: Conception and design, collection and assembly of data, and data analysis and interpretation. Ilaria Zanetti and Gian Luca de Salvo: Data analysis and interpretation. Johannes Merks: Conception and design and data analysis and interpretation.. All authors: Article writing and final approval of the article.

## Supporting information

Risk Stratification for EpSSG non metastatic RMS study

Supplementary Material

## Data Availability

Data subject to third party restrictions The data that support the findings of this study are available from the EpSSG datacenter. Restrictions apply to the availability of these data, which were used under license for this study. Data are available from the authors with the permission of the EpSSG datacenter.
