# Supplementary figures and images for: Local staging and treatment in extremity rhabdomyosarcoma. A report from the EpSSG‐RMS2005 study
Source: Cancer Med. 2020 Sep 1;9(20):7580–9. doi: 10.1002/cam4.3365 (PMC7571832; doi:10.1002/cam4.3365)

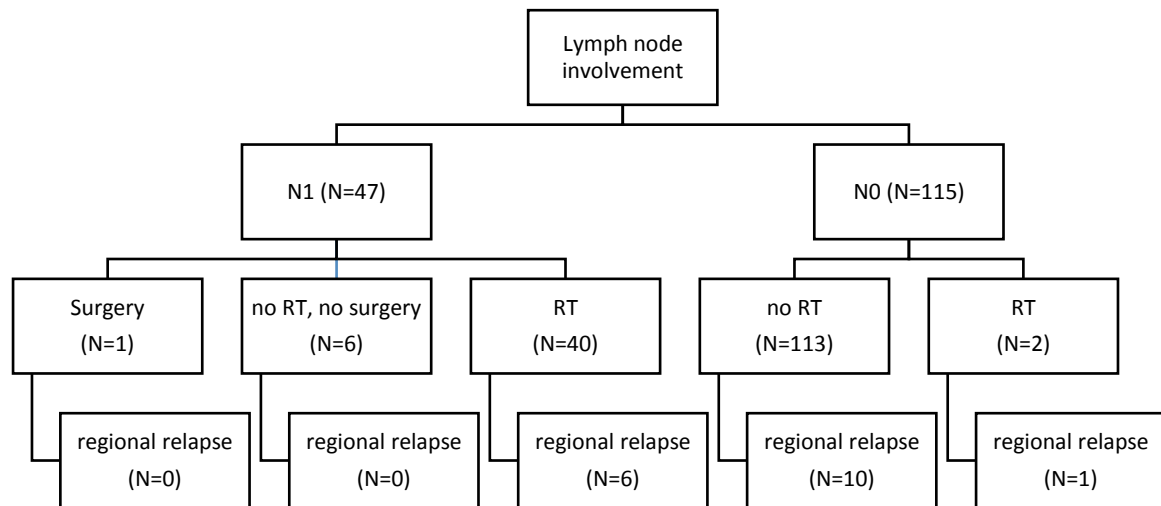

Supplement: Supplementary file 1 — Risk Stratification for EpSSG non metastatic RMS study [file CAM4-9-7580-s001.pdf]
