# Supplementary material for: Local staging and treatment in extremity rhabdomyosarcoma. A report from the EpSSG‐RMS2005 study
Source: Cancer Med. 2020 Sep 1;9(20):7580–9. doi: 10.1002/cam4.3365 (PMC7571832; doi:10.1002/cam4.3365)
Supplement: Supplementary file 2 — Supplementary Material [file CAM4-9-7580-s002.pdf]

Supplemental table 1. Risk Stratification for EpSSG non metastatic RMS study

Risk Group	Subgroups	Pathology	Post surgical Stage (IRS Group)	Site	Node Stage	Size & Age
**Low Risk**	**A**	Favourable	I	Any	N0	Favourable
**Standard Risk**	***B***	Favourable	I	Any	N0	Unfavourable
	C	Favourable	II, III	Favourable	N0	Any
	D	Favourable	II, III	Unfavourable	N0	Favourable
**High Risk**	***E***	Favourable	II, III	Unfavourable	N0	Unfavourable
	F	Favourable	II, III	Any	N1	Any
	G	Unfavourable	I, II, III	Any	N0	Any
**Very High Risk**	***H***	Unfavourable	I, II, III	Any	N1	Any

IRS I, local disease, totally resected. IRS II, macroscopic resection but microscopic residuals, lymph nodes not affected or resected. IRS III, macroscopic residuals and/or lymph nodes affected. N0, lymph nodes not affected. N1 lymph nodes affected
